# Effects of Alternative Solvents in Experimental Enamel Infiltrants on Bond Strength and Selected Properties

**DOI:** 10.1155/2022/4293975

**Published:** 2022-11-14

**Authors:** Tatiany Gabrielle Freire Araújo Guimarães, Victor Pinheiro Feitosa, Tainah Oliveira Rifane, Ravana Angelini Sfalcin, Bruno Martini Guimarães, Americo Bortolazzo Correr

**Affiliations:** ^1^Inapós School of Dentistry, Pouso Alegre, Brazil; ^2^Paulo Picanço School of Dentistry, Fortaleza, Brazil; ^3^Universidade Nove de Julho, São Paulo, Brazil; ^4^Federal University of Alfenas, Alfenas, Brazil; ^5^FOP Unicamp, Piracicaba, Brazil

## Abstract

**Objective:**

To evaluate different concentrations of solvents (tetrahydrofuran (THF) and dimethyl sulfoxide (DMSO) and monomers on the degree of conversion, microtensile bond strength, and mechanical properties of experimental resin infiltrants.

**Materials and Methods:**

Resin infiltrants were formulated and divided into eleven groups: (1) Icon, (2) 75% TEGDMA (T) +25% UDMA (U), (3) T +25% BIS-EMA (B), (4) T + U +0.5%DMSO, (5) T + U +5% DMSO, (6) T + U +0.5% THF, (7) T + U +5% THF, (8) T + B +0.5% DMSO, (9) T + B +5% DMSO, (10) T + B +0.5% THF, and (11) T + B +5% THF. One hundred and ten bovine mandibular incisors were sectioned, treated, and destined to the degree of conversion, tensile cohesive strength, microtensile bond strength, flexural strength, and elastic modulus. Data were submitted to one-way ANOVA and Tukey's test (*α* = 0.05).

**Results:**

The degree of conversion was lowest for T + B +5%THF (41.9%) and highest for T + U +5%THF (62.1%). In flexural strength and E-modulus, the T + B (96.5 MPa and 0.49 GPa) obtained the highest values and the lowest for T + U +5% DMSO (18.5 MPa and 9.7 GPa). Icon showed the highest bond strength (19.3 MPa) and cohesive strength (62.2 MPa), while T + U +5%DMSO (9.7 MPa) and T + B +5% DMSO (9.8 MPa) the lowest values and T + B +0.5% DMSO (12.3 MPa) the lowest cohesive strength.

**Conclusions:**

The addition of lower concentrations of DMSO or THF (0.5%) did not impair bond strength or significantly affect monomer conversion, but reduced the mechanical properties of resin infiltration.

## 1. Introduction

The first signs of dental caries progression and development are shown through white spots and lesions. It indicates the interaction mechanisms between the susceptible host (tooth), diet, microorganism, and time, which culminate in the mineral loss (demineralization) between dental tissues [1, 2]. Moreover, the patient's poor oral hygiene and treatments with the use of retentive devices, as in the case of orthodontic treatment, are considered a prevalence factor in white spot lesions (WSLs) due to the accumulation of bacterial plaque, between 50% and 80% of patients [3, 4].

The early diagnosis of these stains on tooth enamel allows a minimally invasive treatment for the remineralization of active lesions noncavitated [4]. Thus, a treatment option is a remineralization using fluorine or amorphous calcium casein phosphate, which has been shown to positively influence the arrest of early caries [5–7]. However, deeper lesions do not completely remineralize, as the formation of a hypermineralized superficial layer makes mineral deposition in the body of the subsuperficial lesion difficult [8].

For this, a technique using the application of a low-viscosity resin was studied and presented satisfactory laboratory and clinical results [9, 10]. The technique aims to occlude porous structures of incipient enamel lesions through capillarity, to interrupt or delay the advancement of caries processes, enveloping the hydroxyapatite crystals, and micromechanically interlocking the remaining enamel prisms. This material is applied in other cases of fluorosis and dentin hypersensitivity [11]. Therefore, current results of the Icon® material (DMG, Hamburg, Germany) for caries infiltration demonstrate an effective method to prevent the progression of non-cavitated proximal lesions [10, 12].

The main component of the resin infiltrant is trimethylene glycol dimethacrylate (TEGDMA), a low-viscosity, high-fluidity resin monomer that has potentially higher water sorption over time. However, it does not fully seal the enamel only 60% volume of the lesion area [11, 13]. The high hydrophilicity of the monomer in addition to the acidic environment due to bacterial cardiogenesis (high pH) can cause the dissolution of noninfiltrated minerals presence ions (H^+^) [14].

Solvents will be incorporated into resin infiltrant for better volatile properties that may improve the polymer stability and greater penetration in the lesion. Tetrahydrofuran (THF) was recently reported as a viable alternative solvent for an adhesive system [15]. Likewise, dimethylsulfoxide (DMSO) is a solvent widely used in many types of chemical reactions that require high polarity and antimicrobial potential, although it is cytotoxic in large concentrations [16, 17]. The addition of DMSO or THF in infiltration procedures may increase wettability, penetration, and bonding in demineralized enamel as well as exert therapeutic effects, albeit no sufficient report is found in the literature.

Therefore, this study aims at evaluating the influence of solvents at different concentrations on the degree of conversion, bond strength, and elastic modulus of experimental infiltration agents. As a null hypothesis, the combination of different solvents will improve the following: (1) flexural strength and elastic modulus mechanical properties, (2) bond strength, (3) tensile cohesive strength, and (4) degree of conversion.

## 2. Materials and Methods

### 2.1. Preparation of Experimental Infiltrants

The following monomers were used: ethoxylated bisphenol A glycidyl dimethacrylate (BisEMA) (Sigma-Aldrich Inc., Batch #03514HF), urethane dimethacrylate (UDMA) (Sigma-Aldrich Inc., Batch #09405B), and triethylene glycol dimethacrylate (TEGDMA) (Sigma-Aldrich Inc., Batch #01612 M). The solvents dimethyl sulfoxide (DMSO) and tetrahydrofuran (THF) (Sigma-Aldrich Inc., Batch #51496 AM) were in different ratios, as described in Table 1.

The monomers resin blends were mixed up in brown glass jars, for each experimental group. For all blends, the photoinitiator system selected was camphorquinone (CQ) (Sigma-Aldrich, Batch #532604) and dimethyl aminoethyl methacrylate (DMAEMA) (Sigma-Aldrich Inc., Batch #BCBF8391V) as coinitiator (proportion 1 : 2 by weight). The inhibitor butylated hydroxytoluene (BHT) (Sigma-Aldrich Inc., Batch #04416KD) was added to the resin blends with a concentration of 0.1 wt% to avoid the spontaneous polymerization of the monomers. Also, the light-curing initiator system was thoroughly dissolved in the monomer matrix with a concentration of 1.5 wt% (0.5% CQ/1% DMAEMA). To avoid premature polymerization, the resin blend groups were stored at 4°C until use.

### 2.2. Group Division

The division of the groups was established according to the blends composition. (1) Icon, (2) T + B-75% TEGDMA+25% BisEMA, (3) T + B +0.5DMSO-TEGDMA+BISGMA+0.5%DMSO, (4) T + B +5DMSO-TEGDMA+BISGMA+5%DMSO, (5) T + B +0.5THF-TEGDMA+BISGMA+0.5%THF, (6) T + B +5THF-TEGDMA+BISGMA+5%THF, (7) T + U-75% TEGDMA+25%UDMA, (8) T + U +0.5 DMSO-TEGMA+UDMA+0.5%DMSO, (9) T + U+5 DMSO-TEGDMA+UDMA+5% DMSO, (10) T + U +0.5 THF-TEGDMA+UDMA+0.5%THF, and (11) T + U +5THF-TEGDMA+UDMA+5%THF.

### 2.3. Preparation of Enamel Blocks

One hundred and ten bovine mandibular incisors without cracks or caries were collected. The teeth were cleaned and stored in 0.1% thymol solution up to 1 month after extraction. The enamel surfaces were flattened (5 × 5 mm) on a water-cooled mechanical grinding machine using 340- and 600-grit Al_2_O_3_ abrasive paper (Aropol E, Arotec S.A, Ind.&Com., São Paulo, Brazil). The roots were cut 1 mm below the cement enamel junction using a diamond disc (Isomet, Buehler Ltd., Lake Bluff, IL, USA) and discarded. The teeth crowns were cut using a diamond disc (Isomet, Buehler Ltd, Lake Bluff, IL, USA.) to obtain blocks of enamel (5 × 5 mm).

### 2.4. Enamel Demineralization Procedures

The subsurface enamel caries-like lesions (ECLL) were produced on the sound enamel surface. Each enamel block was covered with double coats of acid-resistant nail varnish (Colorama®, São Paulo, Brazil) except for the polished enamel area (5×5 mm). The ECLL was produced by immersion of each enamel surface into 50 mL of a demineralizing solution containing 0.05 M acetate buffer 50% hydroxyapatite saturated from enamel powder, pH 5.0, for 16 h at 37°C. To prepare the solution, enamel powder (particles of 74-105 *μ*m) was agitated into 0.05 sodium acetate buffer, pH 5.0, for 96 h at 37°C (0.50 g/L). The solution was used in a ratio of 2.0 mL/mm^2^ of exposed enamel area. An immersion period of 16 h was determined in a previous study, by analyzing thin enamel slices with polarized light microscopy. Wass observed the presence of subsurface ECLL. Calcium concentration in the solution was 66.3 *μ*g/mL, which was determined by atomic absorption spectrometry with flame spectrophotometer model 506 (Perkin Elmer); phosphorus concentration was about 32 *μ*g/mL, which was determined by colorimetric method with spectrophotometer model 800 M (Analyzer) adjusted at 660 nm.

### 2.5. Specimen Preparation

The enamel blocks with ECLL were randomly distributed into eleven groups (Table 1) (*n* = 10) according to the composition of low viscosity resin materials. The previously determined area (5 × 5 mm) on enamel surface blocks was etched with 37% phosphoric acid gel (Vigodent, Rio de Janeiro, RJ, Brazil) for 60 s rinsed for 10 s, and dried with compressed air for 15 s. The experimental infiltrants were applied using a microbrush for 60 s to improve the penetration into the etched enamel. The Block surface was air dried for 15 s to evaporate the solvent. Infiltrants were then light cured for 60 s using Ultralume 5 (Ultradent, South Jordan, UT, USA) with 1000 mW/cm^2^. Blocks of experimental infiltrants with 4 mm height (increments of 2 mm thickness) were done using a silicon mold, individually light cured for 60 s. Then, enamel blocks were stored in 100% humidity for 24 h at 37°C. Afterward, each enamel block was longitudinally cut into slices of 1 mm thickness, using a water-cooled diamond blade (Isomet, Buehler Ltd, Lake Bluff, IL, USA). Four slices were obtained of each block and trimmed to a dumbbell shape using a cylindrical diamond bur (FF 1092, KG Sorensen, São Paulo, SP, Brazil) in a high-speed handpiece.

### 2.6. Microtensile Bond Strength Test (*μ*TBS)

The dumbbell-shaped specimens were individually fixed to the apparatus and tested using a universal testing machine (EZ-TEST) with a 50 N load at 0.5 mm/min crosshead speed. The cross-sectional area at the site of fracture was measured with a digital caliper (Starrett 727, Starrett Indústria e Comércio LTDA, Itu, Brazil) with an accuracy of 0.01 mm. The microtensile bond strength was calculated in MPa, according to the following formula: *R* = *F* × 0,098/*A*, where *A* is the bonding surface area in cm^2^, *F* is the value of force obtained during the test in kgf, and *R* is the bond strength in MPa [18].

### 2.7. Analysis of Failure Pattern

The fractured specimens were fixed on metallic stubs with double-sided carbon tape (Electron Microscopy Sciences, Washington, USA). The stubs with the fractured specimens were ultrasonically cleaned with distilled water for 10 minutes and dehumidified for 2 hours in an oven (Farnam, Kiln Drying and Sterilization, Model 315 SE, SP, Brazil) at 40°C for 6 hours. All specimens were gold-sputter coated (Balzers model SCD 050 sputter coater, Balzers Union Aktiengesellschaft, Liechtenstein Fürstentum, Germany) at 40 mA for 120 s and observed in Scanning Electron Microscope (SEM) at an accelerating voltage of 15 kV, the working distance (WD) of 33 mm, spot size 44, and magnification of ×50 and ×200. The failure patterns were classified as follows: Type I: cohesive failure in infiltrant; Type II: cohesive failure in enamel; Type III: mixed failure between the infiltrant layer and the enamel. A blind calibrated examiner evaluated the failure pattern. The intraexaminer coincidence level was analyzed by Spearman's correlation, and 95% of coincidence was observed and obtained in a 7 days interval evaluation [19].

### 2.8. Flexural Strength and E-Modulus

Bar specimens (9 mm in length, 2 mm thick, and 2 mm in width) were produced in metallic molds. The specimens (*n* = 10) were light-cured at 1000 mW/cm^2^ at 3 different points each for 60 s using Ultralume 5 (Ultradent, South Jordan, Utah, USA) under a polyester strip (Airon Maquira Dental Products Industry, Maringa, Brazil). Specimens were dry stored for 24 h in light-proof containers at 37°C. To assess the flexural strength and elastic modulus, the three-point-bending test was performed in a universal testing machine (INSTRON, model 4111, Instron Corp., OH, USA). The test was performed with a crosshead speed of 0.5 mm/min and a cell load of 50 N until fracture. The distance between supports was 3 mm; EM was calculated using Bluehill 2 software (Illinois Tool Works Inc., IL, USA) coupled with the universal testing machine [20].

### 2.9. Degree of Conversion

The degree of conversion (DC) of the resin infiltrants commercial was evaluated using the Attenuated Total Reflection Fourier-Transform Infrared spectrophotometer (Nicolet 5700, Thermo Fisher Scientific, Loughborough, UK) equipped with an ATR crystal. The material was dropped on the base of FTIR (*n* = 3), and heights of peaks were collected according to the aliphatic and aromatic peaks.

The same drop (*n* = 3) was light cured (60 s; 1000 mW/cm^2^, Optilux VLC, Demetron Kerr, Orange, USA), and new heights of peaks were collected. The remaining unconverted double bonds were determined by comparing the ratio of the aliphatic *C* = *C* absorption peak at 1638 cm^−1^ to the aromatic group *C* = *C* peak at 1608 cm^−1^ between the polymerized and unpolymerized specimens [21].

### 2.10. Tensile Cohesive Strength

The resin infiltrants (*n* = 10) were inserted into silicon molds to obtain dumbbell shape (2 mm height × 8 mm length × 1.5 mm constriction region) specimens. The material surface was covered with a Mylar strip and then light cured using Ultralume 5 (Ultradent, South Jordan, Utah, USA). The dumbbell-shaped specimens were removed from the molds and polished under irrigation with 600-1200 grit silicon carbide (SiC) abrasive paper. After storage for 24 h, specimens were tested until the failure under tensile using a universal testing machine (EZ Test) with a 50 N load at 0.5 mm/min crosshead speed. The exact area of the transversal section of the fractured specimens was measured with a digital caliper [22].

### 2.11. Statistical Analysis

Data were analyzed using the Shapiro-Wilk normality test and Levene's homoscedasticity (*p* ≤ 0.05). Analysis of variance (ANOVA) followed by the Tukey test. Analyzes were performed using software (SPSS 21, Chicago, IL, USA), and the level of significance was 5% (≤ 0.05).

## 3. Results

The outcomes (means and standard deviations) of the degree of conversion, flexural strength, E-modulus, microtensile bond strength, and tensile cohesive strength are presented in Table 2. Icon® showed the highest bond strength (19.3 MPa), statistically similar to T + U +0.5% DMSO (16.2 MPa) (*p* = 0.0832). Intermediate values are presented by the groups (T + U +0.5%THF, T + U, T + B, T + B +0.5%DMSO, T + U +5%THF, T + B +0.5%THF, and T + B +5%THF (*p* < 0.001)). The addition of 5% DMSO into resin infiltrants significantly decreases the microtensile bond strength.

The flexural strength and E-modulus varied from the lowest for T + U +5% DMSO (18.5 MPa and 9.7GPa, respectively) to the highest for T + B (96.5 MPa and 0.49 GPa, respectively) (*p* < 0.001). The solvent-free experimental infiltrants (T + B and T + U) showed statistically higher flexural strength than other groups. The addition of DMSO on T + U blends significantly decreases flexural strength compared to THF. For T + B blends, there were no significant differences between DMSO and THF. For EM, the ranking was as follows: *T* + *B* ≥ *T* + *B* + 0.5%THF ≥ *T* + *U* + 0.5%THF ≥ *T* + *U* ≥ *T* + *B* + 0.5%DMSO = *T* + *U* + 5%THF ≥ *T* + *B* + 5%THF = *T* + *B* + 5%DMSO  ≥  Icon®≥*T* + *U* + 0.5%DMSO > *T* + *U* + 5%DMSO.

The lowest DC varied from the lowest T + B +5% THF (41.9%) to the highest T + U +5% THF (62.1%) (*p* < 0.001). The blends containing UDMA showed statistically higher degrees of conversion than BisEMA. Icon presented intermediary outcomes (50.4%), significantly higher than T + B +5% DMSO (42.8%), T + B +0.5% THF (42.1%), and T + B +5% THF (41.9%).

**Figure 1:**
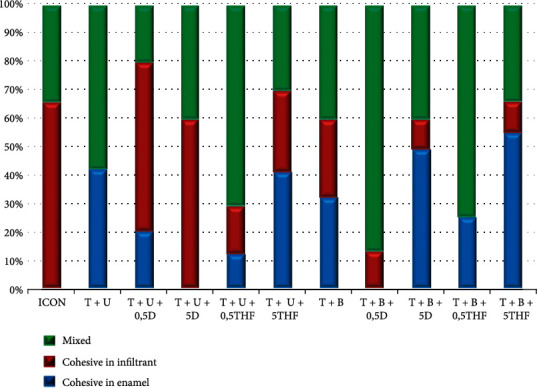
Failure pattern distribution of different groups tested.

Figure 1 shows the graphic illustration of the distribution of fracture patterns observed for each experimental group. The groups T + U, T + U +0.5THF, T + B, T + B +0.5%DMSO, and T + B +0.5% THF presented a mixed predominant fracture pattern. The groups T + U +5%THF, T + B +5% DMSO, and T + B +5%THF showed predominant cohesive failures in enamel. Groups that had the predominant cohesive in infiltrant are T + U + 0.5% DMSO and Icon®.

## 4. Discussion

The solvents assume an important role when it comes to resin infiltration, once solvent-monomer ratios are considered critical factors for its performance. These components serve to facilitate the diffusion and displacement of water on the dentin surface [15, 23]. Therefore, this research adopted THF and DMSO as polar aprotic solvents that do not have a hydroxyl group, which has no participation reaction. The addition of lower solvent concentrations did not impair bond strength or significantly affect the degree of conversion. Thus, the first and fourth hypotheses were accepted, however, reduced the mechanical properties of the resin infiltration, rejecting the second and third hypotheses.

In the bond strength test, the Icon and T + U +0.5DMSO groups were statistically higher. It may be explained by the fact that DMSO is a polar solvent with the capacity to dissolve a wide variety of substrates, including organic molecules, carbohydrates, polymers, peptides as well as inorganic salts, and gases. This mechanism modifies the interaction between dentin and resin infiltrant monomers influencing its diffusion [24, 25]. Its molecule has a group (*S* = *O*) and two hydrophobic methyl groups highly polar with hydrophilic features, miscible with most commonly used monomers [25–27]. DMSO makes hydrogen bonds breaking its self-associating ability in 0.5% concentrations as shown in this research, increasing the bond strength. These amphiphilic features also influence the enamel surface wettability and the resin infiltrant penetration in noncavitated carious lesions [28, 29].

Fracture analysis of T + U +5%THF, T + B +5%DMSO, and T + B +5%THF was classified as cohesive in enamel. Therefore, the solvent must be evaporated after performing its functions as it may compromise the polymerization and trigger the early bonding interface degradation due to the presence of residual moisture [26, 29, 30]. The rate of evaporation of any substance is determined by a property of liquids called vapor pressure. Those that evaporate faster have a higher vapor pressure. DMSO has a lower vapor pressure, higher boiling pressure, and low viscosity, making it difficult to volatile especially in large concentrations when compared to THF [29, 31]. The heterocyclic organic solvent THF has polar features able to dissolve polar and nonpolar components, with high vapor pressure and greater volatilization than absolute ethanol, which may be efficient to remove water from the enamel, before the resin infiltrant application [31]. The evaporation is shown to get greater results when THF is applied with resin infiltrant, once the remaining water may still be in the enamel, providing an adequate material infiltration using a simpler technique compared to ethanol [29].

In the present study, the addition of DMSO or THF did not significantly affect the degree of conversion, for all amounts and types of solvents added to the mixture, with statistically similar results. The monomeric composition and its relation to solvent play the main role in the polymerization behavior. The solvents allow the displacement of water that must be eliminated from the bonding surfaces [28] besides causing a mixture of hydrophilic and hydrophobic monomers. When a polymer comes into contact with solvents, chemical bonds are formed between the solvent and polar groups present in the polymer structure (e.g., OH and CO). DMSO has two nonpolar methyl groups (CH3) that interact with hydrophobic portions of BisEMA/UDMA [27] and the structure of THF. The mobility of reactive monomer components during cure increases the conversion of monomers [29]. Furthermore, DMSO also reduces termination rates in free radical polymerization methacrylate, as it does not evaporate, being a positive feature to avoid the separation phase [28, 30]. The monomer conversion after the polymerization of light-curing materials affects mechanical properties, such as tensile strength, compression and bending, elastic modulus, wear, and hardness.

Furthermore, the icon did not obtain a value higher than 50% monomer conversion as already shown in a previous study [32] and the present study. The presence of TEGDMA, a hydrophilic monomer, is susceptible to water sorption and hydrolysis. In addition, the oxygen-inhibited layer results in a low polymerization rate, negatively influencing some mechanical properties [10, 17]. Thus, the association of solvents could be an alternative to improve the degree of conversion in this study which showed > 60% conversion but with some reduced mechanical properties which may affect its clinical performance to prevent the progression of noncavitated caries lesions.

There were significant differences between T + B in flexural strength and modulus of elasticity. T + U had higher results, and DMSO and THF had lower results mainly at 5% concentration. The groups containing different solvent concentrations affected this mechanical property, as in the strength of the cohesive bond. Solvents were added to the resin infiltrant to evaporate the remaining water that might still be in the enamel. However, it is noted that solvent residues even at the lowest concentrations were able to affect the material's mechanical strength. After performing its function, it is not sufficiently evaporated, resulting in dilution of monomers and phase separation of resin components [33, 34]. This scenario causes component emulsion, separating into hydrophobic and hydrolytic phases. Additionally, incomplete polymerization also leads to greater polymer permeability; in some cases leaching of residual monomers from dental resins may cause local or systemic allergic or poisonous effects [26, 29, 30, 35].

After all, the solvents were added to the base monomers composition to provide its miscibility, resin infiltrant diffusion in the enamel, and rapid volatilization after application. The results showed that the mixtures composed of BisEMA presented a significantly lower degree of conversion than those based on UDMA. This may be related to the higher molecular weight (629 g/mol) of BisEMA and UDMA (470 g/mol). Its lower chain flexibility may be linked to benzene rings in the middle of the chain. Moreover, its reactivity during polymerization was lower [31, 36]. The UDMA monomer has flexible chains and two aliphatic urethane bonds that are capable of forming hydrogen bonds. This may also function as a tertiary amine, which explains the monomer conversion values statistically higher than the infiltrant containing BISEMA. TEGDMA is a highly flexible low molecular weight and low viscosity monomer that decreases the viscosity of high molecular weight monomers, which contribute to mobility during polymerization. However, fewer resistant polymers are formed after polymerization due to its hydroxyls molecules. The greater its quantity, the greater the capacity to make hydrogen bonds with water, increasing the material's sorption and solubility and decreasing its mechanical properties [31, 33, 36, 37].

Therefore, the mechanical properties of the resin infiltrants were reduced in all groups when the solvent percentage increased from 0.5% to 5%, for both DMSO and THF, without affecting the degree of conversion.

## 5. Conclusions

Within the limitations, we conclude that the addition of lower concentrations of DMSO or THF (0.5%) did not impair bond strength or significantly affect the degree of conversion, nevertheless reducing the mechanical properties of the resin infiltration. The greater results were shown by blends containing UDMA and TEGDMA.

## Figures and Tables

**Table 1:** Infiltrant blends composition.

Infiltrant	Composition (wt%)
Icon (DMG, Hamburg, German, batch number: 633139)	Icon-etch: hydrochloric acid, pyrogenic silicic acid, and surface-active substances. Icon-dry: 99% ethanol Icon-infiltrant: TEGDMA-based resin matrix, initiators, and additives
T + B	75% TEGDMA+25% BisEMA
T + B +0.5DMSO	TEGDMA+BISGMA+0.5%DMSO
T + B +5DMSO	TEGDMA+BISGMA+5%DMSO
T + B +0.5 THF T + B +5 THF	TEGDMA+BISGMA+0.5%THF TEGDMA+BISGMA+5%THF
T + U	75% TEGDMA+25%UDMA
T + U +0.5 DMSO	TEGMA+UDMA+05%DMSO
T + U +5 DMSO	TEGDMA+UDMA+5%DMSO
T + U +0.5 THF	TEGDMA+UDMA+0.5%THF
T + U +5 THF	TEGDMA+UDMA+5%THF

T: triethyleneglycol dimethacrylate; B: ethoxylated bisphenol A glycidyl dimethacrylate; U: urethane dimethacrylate; DMSO: Dimethyl Sulfoxide; THF: tetrahydrofuran.

**Table 2:** Mean (standard deviation) values of degree of conversion, flexural strength, elastic modulus, bond strength, and tensile cohesive strength of resin infiltrants.

Groups	Degree of conversion (%)	Flexural strength (MPa)	Elastic modulus (GPa)	Bond strength (MPa)	Tensile cohesive strength (MPa)
Icon®	50.4 (1.6) b	62.4 (20.3) bc	0.28 (0.10) de	19.3 (1.4) a	62.2 (2.0) a
T + B	46.8 (1.9) bcd	96.5 (20.9) a	0.49 (0.10) a	14.3 (1.4) b	28.3 (6.6) cd
T + B +0.5DMSO	47.9 (1.4) bc	64.8 (15.6) bc	0.35 (0.12) bcde	14.1 (1.2) b	12.3 (1.9) e
T + B +5DMSO	42.8 (0.8) cd	64.6 (4.9) bc	0.28 (0.05) cde	9.8 (1.9) c	22.6 (0.9) d
T + B +0.5THF	42.1 (3.2) d	85.8 (14.4) b	0.48 (0.10) ab	13.8 (0.7) b	39.6 (4.1) bc
T + B +5THF	41.9 (3.9) d	70.9 (17.8) b	0.32 (0.04) cde	13.6 (0.8) b	30.0 (5.1) bcd
T + U	61.2 (0.4) a	96.1 (20.4) a	0.39 (0.10) abcd	14.4 (1.8) b	30.0 (5.1) bcd
T + U +0.5DMSO	61.9 (0.2) a	45.7 (10.6) c	0.25 (0.07) e	16.2 (1.4) ab	37.6 (15.2) bc
T + U +5DMSO	61.5 (0.2) a	18.5 (6.2) d	0.07 (0.02) f	9.7 (1.8) c	38.2 (7.3) bc
T + U +0.5THF	61.2 (0.2) a	83.7 (18.5) b	0.41 (0.08) abc	16.1 (1.6) b	40.9 (2.9) bc
T + U +5THF	62.1 (0.3) a	61.2 (17.8) bc	0.34 (0.10) bcde	14.0 (0.9) b	42.3 (4.0) b

Different letters in columns indicate statistically significant differences (*p* < 0.05). T: triethyleneglycol dimethacrylate; B: ethoxylated bisphenol A glycidyl dimethacrylate; U: urethane dimethacrylate (UDMA); DMSO: dimethyl sulfoxide; THF: tetrahydrofuran.

## Data Availability

Experimental data are available upon request.
